# A Proteomic View of the Cross-Talk Between Early Intestinal Microbiota and Poultry Immune System

**DOI:** 10.3389/fphys.2020.00020

**Published:** 2020-02-13

**Authors:** D. R. Rodrigues, K. M. Wilson, M. Trombetta, W. N. Briggs, A. F. Duff, K. M. Chasser, W. G. Bottje, L. Bielke

**Affiliations:** ^1^Department of Animal Sciences, The Ohio State University, Columbus, OH, United States; ^2^Department of Poultry Science, University of Arkansas, Fayetteville, AR, United States

**Keywords:** segmented filamentous bacteria, probiotic *in ovo*, immunity, inflammation, *Enterobacteriaceae*, pioneer colonizers, Ingenuity Pathway Analysis

## Abstract

Proteomics has been used to investigate cross-talk between the intestinal microbiome and host biological processes. In this study, an *in ovo* technique and a proteomics approach was used to address how early bacterial colonization in the gastrointestinal tract (GIT) could modulate inflammatory and immune responses in young broilers. Embryos at 18 embryogenic days were inoculated with saline (S), 10^2^ CFU of *Citrobacter freundii* (CF), *Citrobacter* species (C2), or lactic acid bacteria mixture (L) into the amnion. At 10 days posthatch, ileum samples from 12 birds per treatment were selected for tandem mass spectrometry analysis. Our further findings indicated that treatment-specific influences on early GIT microbiota resulted in different immune responses in mature broilers. Predicted functional analyses revealed activation of inflammation pathways in broilers treated *in ovo* with L and CF. Exposure to L enhanced functional annotation related to activation, trafficking of immune cells, and skeletal growth based-network, while CF inhibited biological functions associated with immune cell migration and inflammatory response. These results highlighted that proper immune function was dependent on specific GIT microbiota profiles, in which early-life exposure to L-based probiotic may have modulated the immune functions, whereas neonatal colonization of *Enterobacteriaceae* strains may have led to immune dysregulation associated with chronic inflammation.

## Introduction

Pioneer colonization of intestinal microbiota has a major effect on driving the maturation and composition course of microbial communities over time (Juricova et al., 2013; Rodrigues et al., 2019; Wilson et al., 2019). Later, the cross-talk between microbiota composition and immune cells has been highly associated with the establishment of immune competence (Crhanova et al., 2011; Chung et al., 2012; Schokker et al., 2017; Duan, 2018). Germ-free mouse models have been essential to reveal a strong influence of intestinal microbial communities on the proper immune function. The lack of intestinal microbiota in these mice caused extensive deficits in the development of the gut-associated lymphoid tissues, abnormal production of immune cells, and other immunological deficiencies (Round and Mazmanian, 2009). In this context, a recent study with broilers has shown that the use of antibiotics during early life perturbed microbiota colonization, subsequently triggering an alteration in systemic immune programming (Schokker et al., 2017). Nevertheless, the specific microbial populations involved in immune-modulatory functions are beginning to be deciphered with the advancement of metagenomic analyses.

It has been reported by Kogut (2019) that the avian neonatal phase is an important window of opportunity to manipulate the intestinal microbiome toward beneficial bacterial growth. In fact, our previous studies showed that early exposure of embryos to lactic acid bacteria or *Enterobacteriaceae* strains resulted in different microbiome profiles at day of hatch and 10 days of age, suggesting that neonatal exposure to beneficial bacteria may be critical for influencing gastrointestinal tract (GIT) populations throughout the maturation of the poultry microbiota (Wilson et al., 2019). However, whether this pioneer intestinal microbiome modulation can affect the host immunological functions remain unclear.

In this study, the influence of early intestinal bacterial colonization on the inflammatory and immune response of young broilers was investigated. For this purpose, two non-pathogenic *Enterobacteriacea*e isolates and a lactic acid bacteria-based probiotics were introduced *in ovo*, and mass spectrometry-based proteome analysis was performed on ileum tissue. To test our hypothesis, we focused on intestinal inflammatory and immune-related proteins, screened the biological functions predicted by Ingenuity Pathway Analysis (IPA), and linked inflammation biomarkers to intestinal microbial signatures established in the ileal microbiome of 10-day-old broiler chickens.

## Materials and Methods

### Experimental Design

**FIGURE 1:**
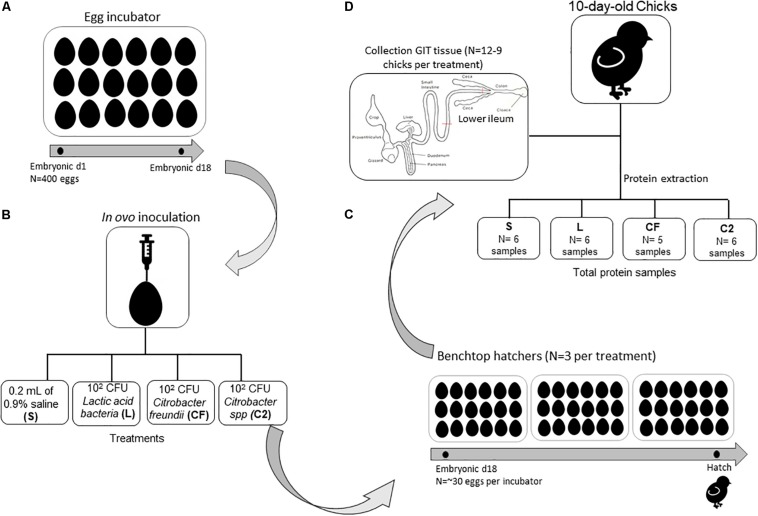
Schematic overview of experimental design and gastrointestinal (GIT) tissue collection for proteome analyses. (**A**) Four hundred eggs were incubated under standard conditions in one single-stage egg incubator until embryonic day 18. (**B**) *In ovo* inoculations contained one of the following: 0.2 ml of 0.9% sterile saline (S), which served as the control group, or ∼10^2^ cells of *Citrobacter freundii* (CF), *Citrobacter* spp. (C2), or a lactic acid bacteria mixture (L) was administered into the amnion. (**C**) After inoculation, up to 30 eggs were allocated by treatments into three separate benchtop hatchers per treatment. (**D**) Ten days posthatch, chicks were selected for ileum sample collection (pooled samples of two birds per treatment) to perform proteome analyses.

A total of 400 eggs from commercial Ross 708 broiler breeder flocks were obtained from a local hatchery. Per standard operating procedures, the eggs were sanitized before storage and incubation. All eggs were incubated under standard conditions at the Ohio Agricultural Research and Development Center’s poultry research farm. Once eggs were confirmed fertile, at embryonic day 18, the air-cell end of each egg was treated with iodine (povidone–iodine 10% topical solution, Drug Mart, Medina, OH, United States) before a small hole was punched into the shell with an inoculation needle. *In ovo* inoculations contained one of the following: 0.2 ml of 0.9% sterile saline (S), which served as the control group, or approximately 10^2^ cells of *Citrobacter freundii* (CF), *Citrobacter* spp. (C2), or lactic acid bacteria mixture (L) administered into the amnion (Figure 1). After inoculation, up to 30 eggs were allocated by treatments into three separate benchtop hatchers (Hova-Bator model 1602N, Savannah, GA, United States) for a total of 12 hatchers. All hatchers were disinfected with 10% bleach before use. Strains CF and C2 were selected from our previous study as non-pathogenic bacteria from the gut of healthy birds (Bielke et al., 2003), and the homology of strains was confirmed by next-generation sequencing. The L culture was composed of a mixed inoculum of *Lactobacillus salivarius* and *Pediococcus* sp. Bacterial inoculations were prepared as described by Wilson et al. (2019). Preliminary experimental observations concluded that the inclusion of isolates at ∼10^2^ CFU did not affect hatchability compared to the S control treatment (data not published). All experimental procedures were approved by the Ohio State University’s Institutional Animal Care and Use Committee.

### Sample Collection

Immediately posthatch, chicks were comingled on a treatment basis, and 128 chicks were placed into treatment-separated brooder battery cages with *ad libitum* access to a standard corn–soy diet and water (Nutrient Requirements of Poultry, 1994). At 10 days posthatch, 12 chicks per treatment were randomly selected for ileal proteome analysis, however, only nine birds were sampled from CF. Chicks were euthanized via cervical dislocation, and the region proximal to the ileocecal junction and distal to Meckel’s diverticulum, designated as lower ileum, was aseptically collected *post mortem* (Figure 1). Ileum tissue was placed into 1.5-ml tubes, flash frozen in liquid nitrogen at the time of collection, and stored at −80°C until further use.

Once thawed, 0.1 g of ileal tissue from each sample was individually placed in 5 ml of buffer (8 M urea/2 M thiourea, 2 mM dithiothreitol, 50 mM Tris, 5% sodium dodecyl sulfate). The extraction protocol was a modified version previously described by Iqbal et al. (2004) and Kong et al. (2016). In brief, samples were homogenized for 5 s (PRO250 Homogenizer, Pro Scientific, Oxford, CT, United States), then 500 μl of homogenate was added to 2-ml tubes containing 0.1 g stainless steel beads (SSB14B Next Advance, Averill Park, NY, United States). Samples were homogenized for a total of 3 min in 30-second intervals (MiniBeadbeater-16, Model 607, BioSpec Products, Bartlesville, OK, United States) and centrifuged at 4°C at 14,000 rpm (21,952 × *g*) for 20 min. The supernatant was collected, aliquoted, and placed into −80°C until further use.

To ensure proper extraction, concentration of total protein was quantified with the Bradford assay (Bradford reagent, VWR, Suwanee, GA, United States) and a standard bovine serum albumin curve (VWR, Suwanee, GA, United States) on a Synergy HTX multimode plate reader (BioTek U.S., Winooski, VT, United States). Samples were mixed to create pooled samples of two birds per treatment (*n* = 6 samples for L and C2; *n* = 5 samples CF; Figure 1) and sent to the Ohio State University Proteomics Core lab for in-solution digestion and mass spectrometry.

### Proteomics Analyses

Samples were precipitated with trichloroacetic acid and then resuspended in 50 mM ammonium bicarbonate. A total of 5 ml of dithiothreitol (5 μg/μl in 50 mM ammonium bicarbonate) was added, and the samples were incubated at 56°C for 15 min. After incubation, 5 μl of iodoacetamide (15 mg/ml in 50 mM ammonium bicarbonate) was added, and the samples were kept in the dark at room temperature for 30 min. Sequencing grade-modified trypsin (Promega; Madison, WI, United States) prepared in 50 mM ammonium bicarbonate was added to each sample at an estimated 1:20/1:100 enzyme/substrate ratio and incubated at 37°C overnight. The reaction was quenched the following day by adding acetic acid for acidification. Once samples were quenched, the peptide concentration was measured by Nanodrop (Thermo Scientific Nanodrop 2000; Waltham, MA, United States).

Capillary-liquid chromatography-nanospray tandem mass spectrometry (capillary-LC/MS/MS) of global protein identification was performed on a Thermo Fisher Fusion mass spectrometer (Thermo Scientific, Waltham, MA, United States). Samples were separated on a Thermo Nano C18 column (UltiMate^TM^ 3000 HPLC system, Thermo Scientific; Waltham, MA, United States). The MS/MS data sequences were scanned and based on the preview mode data-dependent TopSpeed^TM^ method with collision-induced dissociation and electron-transfer dissociation as fragmentation methods. The raw data were searched on Sequest via Proteome Discoverer (Proteome Discoverer^TM^ software, Thermo Scientific, Waltham, MA, United States). The data were searched against the most recent Uniprot *Gallus gallus* database for the identification of proteins. Only proteins with <0.05 false discovery rate were reported. Proteins with a Mascot score of 50 or higher with a minimum of two unique peptides from one protein having a −b or −y ion sequence tag of five residues or better were accepted. Any modifications or low-score peptide/protein identifications were manually checked for validation.

### Biological Interpretation

Label-free quantitation was performed using the spectral count approach, in which the relative protein quantitation is measured by comparing the number of MS/MS spectra identified from the same protein in each of the multiple LC/MS/MS datasets. Comparisons between *in ovo* bacterial treatments and S control group were performed in Scaffold (Scaffold 4.8.4, Proteome Software, Portland, OR, United States). Student’s *t* test (*p* < 0.05) was performed to identify significance across the fold-change values. Differentially expressed proteins (DEPs) at the level of *p* ≤ 0.10 were uploaded into IPA system^1^ to retrieve further inflammatory and immune information in terms of gene ontology, upstream regulators, and causal networks. The IPA functionalities for differentially expressed genes in chicken are based primarily on mammalian biological mechanisms (Kong et al., 2011). The statistical measure *Z* score was displayed to make predictions about potential activation (*Z* score ≥ 2.00) or inhibition (*Z* score < −2.00) of regulators using the information of the protein regulation direction. Qualified predictions were also made for high (*Z* score ≥ | 1.90| or more) medium (*Z* score = | 1.70–1.90|) or low (*Z* score = | 1.50–1.70|), respectively (Kong et al., 2016). Casual networks were performed to show mechanistic hypotheses to explain the expression changes observed in the datasets based on cause–effect relationships reported in the literature (Krämer et al., 2014). The *p* value of the overlap, which measures any significant statistical overlap between the samples in the dataset and the genes that are regulated by the corresponding transcriptional regulator, was also determined and recorded. Fisher’s exact test calculated the value at a significance of *p* < 0.05.

### Correlation Analyses

To further identify the specific bacterial groups that primarily accounted for the differences observed in the expression of inflammation-related proteins in the ileum of broilers, the microbiome data of same samples were added into the analysis, from BioProject ID PRJNA552855, previously published by Rodrigues et al. (2019). The most dominant genus-level operational taxonomic units detected in the four treatments were identified (Supplementary Table S1). Based on the inflammatory annotation generated by the IPA system, the DEPs involved in the inflammation signaling also were determined. Given the considerable number of variables, only overexpressed proteins or those with at least one upregulated DEP within each treatment dataset were selected. Owing to the unlikelihood of perfectly linear relationships and the presence of significant variation between relative bacterial abundance and protein log2-fold change, we applied Spearman’s correlation coefficient (*R*) using RStudio software.

## Results

**FIGURE 2:**
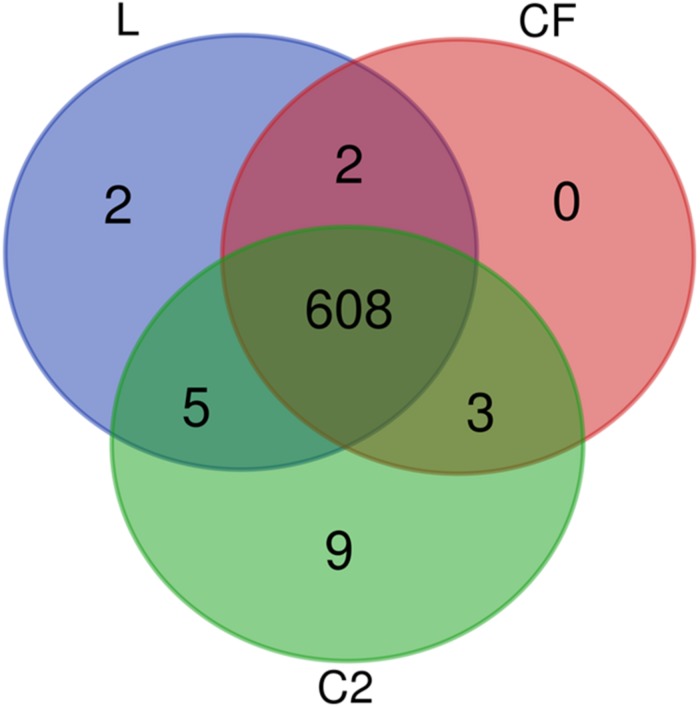
Venn diagram depicting unique and shared proteins identified in the ileum of 10-day-old broilers treated *in ovo* with lactic acid bacteria mixture (L), *Citrobacter freundii* (CF), or *Citrobacter* spp. (C2).

From the proteomic datasets, we identified 617 proteins in L, 613 proteins in CF, and 625 proteins in C2 (Supplementary Tables S2–S4, respectively). Accordingly, 608 proteins were common across all three treatment conditions (Figure 2).

Subsequently, DEPs significantly lower or equal to 0.1 were identified. A total of 61 DEPs were displayed in L (Table 1), 44 DEPs in CF (Table 2), and 63 in C2 (Table 3). Furthermore, we only evaluated the biological interactions associated with inflammatory and immune response signaling.

**TABLE 1:** Differentially expressed proteins (DEPs) from ileal samples of broilers treated with lactic acid bacteria mixture (L) *in ovo.*

**UniProt ID**	**Proteins**	**Protein name**	**Fold change**	***p* Value**
Q10751	ACE	Angiotensin I converting enzyme	–1.000	0.003
P02612	MYL9	Myosin light chain 9	2.400	0.004
Q5ZIV5	PDCD10	Programmed cell death 10	6.800	0.005
P20678	Cyp2c23	Cytochrome P450, family 2, subfamily c, polypeptide 23	–10.000	0.007
Q9PW72	PDLIM4	PDZ and LIM domain 4	–2.000	0.013
P24797	ATP1A2	ATPase Na + /K + transporting subunit alpha 2	–1.600	0.014
P50594	MAGOH	Mago homolog, exon junction complex subunit	4.400	0.015
Q90597	MX2	MX dynamin like GTPase 2	–1.600	0.016
P08940	LECT2	Leukocyte cell derived chemotaxin 2	3.500	0.017
P67883	RPL30	Ribosomal protein L30	–1.660	0.019
Q5ZMU6	PPP4R2	Protein phosphatase 4 regulatory subunit 2	∞	0.021
P31696-10	AGRN	Agrin	–5.000	0.024
P02701	AVD	Avidin	6.700	0.027
E1C2P3	HSPA14	Heat shock protein family A (Hsp70) member 14	5.000	0.029
P05180	Cyp2c23	Cytochrome P450, family 2, subfamily c, polypeptide 23	–3.300	0.031
P47990	XDH	Xanthine dehydrogenase	–2.500	0.031
Q5ZIW2	CNOT10	CCR4-NOT transcription complex subunit 10	–3.300	0.032
P05122-5	CKB	Creatine kinase B	3.300	0.033
P02789	TF	Transferrin	–1.250	0.035
O57535	NME2	NME/NM23 nucleoside diphosphate kinase 2	–1.420	0.037
Q6IVA4	UBA5	Ubiquitin like modifier activating enzyme 5	3.500	0.038
Q5ZKG5	ACP1	Acid phosphatase 1	–2.000	0.041
Q07598	SCP2	Sterol carrier protein 2	–2.000	0.042
Q5ZKP6	ADA	Adenosine deaminase	–2.000	0.043
P09540	MYL4	Myosin light chain 4	1.800	0.045
Q5ZLN5	TARDBP	TAR DNA binding protein	–2.000	0.046
P08636	RPS17	Ribosomal protein S17	1.300	0.046
P61160	ACTR2	ARP2 actin related protein 2 homolog	–1.250	0.051
Q5ZJL9	SAMHD1	SAM and HD domain deoxynucleoside triphosphate triphosphohydrolase 1	2.600	0.052
Q5ZK88	PSPC1	Paraspeckle component 1	–1.660	0.053
Q90629	PRSS2	Serine protease 2	–2.500	0.054
Q02391	GLG1	Golgi glycoprotein 1	–1.600	0.055
P14105	MYH9	Myosin heavy chain 9	–1.250	0.060
P84173	PHB	Prohibitin	–1.420	0.063
P26990	ARF6	ADP ribosylation factor 6	2.400	0.063
P47807	MYO1A	Myosin IA	–1.600	0.066
Q5ZLN1	PGAM1	Phosphoglycerate mutase 1	–1.420	0.068
Q5ZHX7	CYB5R2	Cytochrome b5 reductase 2	–2.500	0.072
Q5ZLR5	UQCRFS1	Ubiquinol-cytochrome c reductase, Rieske iron–sulfur polypeptide 1	1.400	0.074
P17790	BSG	Basigin (Ok blood group)	2.100	0.075
Q9YH06	HMGB1	High mobility group box 1	1.700	0.078
Q5ZLT0	XPO7	exportin 7	–2.000	0.084
Q5ZKD7	MOV10	Mov10 RISC complex RNA helicase	–1.420	0.084
P27003	S100A10	S100 calcium binding protein A10	∞	0.084
P10184	SPINK5	Serine peptidase inhibitor, Kazal type 5	–2.000	0.085
P05083	ASL	Argininosuccinate lyase	2.200	0.085
P23228	HMGCS1	3-Hydroxy-3-methylglutaryl-CoA synthase 1	–2.500	0.086
Q5ZM33	HP1BP3	Heterochromatin protein 1 binding protein 3	1.500	0.086
Q5ZK62	ACAP2	ArfGAP with coiled coil, ankyrin repeat PH domains 2	–1.000	0.093
P16924	P4HA1	Prolyl 4-hydroxylase subunit alpha 1	–1.420	0.095
Q5ZKV8	KIF2A	Kinesin family member 2A	3.500	0.095
O42265	PSMA1	Proteasome subunit alpha 1	–1.660	0.097
P68139	ACTA1	Actin, alpha 1, skeletal muscle	3.200	0.098
Q90615	ITGA1	Integrin subunit alpha 1	1.800	0.099
Q5ZHP5	CHMP4B	Charged multivesicular body protein 4B	–1.420	0.100
P70079	CKMT1	Creatine kinase U-type, mitochondrial	–1.250	0.100
P09652	TUBB3	Tubulin beta 3 class III	2.500	0.100
P24802	PLOD1	Procollagen-lysine,2-oxoglutarate 5-dioxygenase 1	3.000	0.100
Q5ZKC1	EIF2A	Eukaryotic translation initiation factor 2A	∞	0.100

**TABLE 2:** Summary of differentially expressed proteins (DEPs) identified in the ileum of broilers exposed to *Citrobacter freundii* (CF) *in ovo.*

**UniProt ID**	**Proteins**	**Protein name**	**Fold change**	***p* Value**
Q6JHU8	P3H1	Prolyl 3-hydroxylase 1	3.200	0.003
Q5ZKC9	YWHAZ	Tyrosine 3-monooxygenase/tryptophan 5-monooxygenase activation protein zeta	1.500	0.003
Q5ZI89	TRAPPC11	Trafficking protein particle complex 11	–1.000	0.007
Q01841	TGM2	Transglutaminase 2	–1.420	0.010
P00940	TPI1	Triosephosphate isomerase 1	1.500	0.015
Q10751	ACE	Angiotensin I converting enzyme	–10.000	0.016
P55080	MFAP1	Microfibril-associated protein 1	7.500	0.018
Q5ZIV5	PDCD10	Programmed cell death protein 10	5.800	0.021
Q9PW72	PDLIM4	PDZ and LIM domain 4	–5.000	0.021
Q5ZM35	TWF2	Twinfilin actin binding protein 2	7.100	0.022
Q90615	ITGA1	Integrin subunit alpha 1	1.600	0.026
Q02391	GLG1	Golgi glycoprotein 1	–4.000	0.028
Q5ZLN0	LRRC40	Leucine rich repeat containing 40	2.200	0.031
Q5ZJ08	YARS	Tyrosyl-tRNA synthetase	–4.000	0.031
Q02020	FGB	Fibrinogen beta chain	–1.600	0.032
P26446	PARP1	Poly(ADP-ribose) polymerase 1	–3.300	0.036
Q9W6H0	OGN	Osteoglycin	1.600	0.037
Q5ZKU5	RAB14	RAB14, member RAS oncogene family	–4.000	0.038
P51890	LUM	Lumican	–2.500	0.042
Q5ZHN4	RP2	RP2, ARL3 GTPase activating protein	–3.300	0.051
P00548	PKM	Pyruvate kinase M1/2	–1.250	0.052
P0CB50	PRDX1	Peroxiredoxin 1	–1.600	0.052
P19966	TAGLN	Transgelin	2.100	0.057
P87362	BLMH	Bleomycin hydrolase	–2.500	0.059
Q08392	Gsta1	Glutathione S-transferase alpha 1	1.700	0.059
P13914	NAT1	*N*-acetyltransferase 1	2.600	0.059
Q5ZIY5	PPP2R2D	Protein phosphatase 2 regulatory subunit B delta	2.000	0.062
P05083	ASL	Argininosuccinate lyase	2.200	0.064
P23228	HMGCS1	3-Hydroxy-3-methylglutaryl-CoA synthase 1	–3.300	0.064
Q90YH9	TES	Testin LIM domain protein	–2.500	0.067
Q5F381	EPCAM	Epithelial cell adhesion molecule	–1.420	0.069
P56517	HDAC1	Histone deacetylase 1	–4.000	0.070
A0A1N8W591	HDAC1	Histone deacetylase	1.200	0.075
Q90611	MMP2	Matrix metallopeptidase 2	–1.000	0.076
Q5F418	PSMD1	Proteasome 26S subunit, non-ATPase 1	–4.000	0.076
Q5ZIL2	VPS29	VPS29, retromer complex component	–1.000	0.076
Q5F464	LPP	LIM domain containing preferred translocation partner in lipoma	1.300	0.079
Q5ZJ27	HOOK1	Hook microtubule tethering protein 1	–1.000	0.090
P02701	AVD	Avidin	6.200	0.099
Q05744	CTSD	Cathepsin D	1.800	0.100
P12902	HMG-14A	Non-histone chromosomal protein HMG-14A	–3.300	0.100
Q98TF8	RPL22	Ribosomal protein L22	2.000	0.100

**TABLE 3:** Differentially expressed proteins (DEPs) in ileum of 10-day-old broilers exposed to *Citrobacter* spp. (C2) *in ovo.*

**UniProt ID**	**Proteins**	**Protein name**	**Fold change**	***p* Value**
P05122	CKB	Creatine kinase B	–1.250	0.002
P47836	RPS4X	Ribosomal protein S4 X-linked	–1.600	0.002
Q2IAL7	CATHL2	Cathelicidin-2	4.500	0.003
P05122-5	CPK-B	Creatine kinase B Isoform 4	–10.000	0.006
P09652	TUBB3	Tubulin beta 3 class III	2.900	0.006
Q9YGQ1	EEF1B2	Eukaryotic translation elongation factor 1 beta 2	1.800	0.008
Q5ZMU6	PPP4R2	Protein phosphatase 4 regulatory subunit 2	∞	0.016
P24802	PLOD1	Procollagen-lysine,2-oxoglutarate 5-dioxygenase 1	3.400	0.020
Q5ZHP5	CHMP4B	Charged multivesicular body protein 4B	–1.600	0.021
Q5ZIW2	CNOT10	CCR4-NOT transcription complex subunit 10	–5.000	0.028
Q5ZKA4	EIF3J	Eukaryotic translation initiation factor 3 subunit J	1.600	0.032
Q90615	ITGA1	Integrin subunit alpha 1	1.600	0.032
Q8UVD9	KHSRP	Far upstream element-binding protein 2	–1.420	0.034
P05083	ASL	Argininosuccinate lyase	2.300	0.035
Q5ZHN4	RP2	RP2, ARL3 GTPase activating protein	–3.300	0.037
Q5ZKC1	EIF2A	Eukaryotic translation initiation factor 2A	∞	0.039
P41125	RPL13	Ribosomal protein L13	1.700	0.041
Q5ZLR5	UQCRFS1	Ubiquinol-cytochrome c reductase, Rieske iron-sulfur polypeptide 1	1.600	0.044
P27731	TTR	Transthyretin	1.800	0.049
Q5ZLN1	PGAM1	Phosphoglycerate mutase 1	–1.420	0.050
P23228	HMGCS1	3-Hydroxy-3-methylglutaryl-CoA synthase 1	–3.300	0.051
P08106	HSPA2	Heat shock protein family A (Hsp70) member 2	–1.420	0.051
Q5ZK33	LETM1	Leucine zipper and EF-hand containing transmembrane protein 1	2.400	0.051
P49712	ATP6V1B2	ATPase H + transporting V1 subunit B2	1.600	0.052
Q5ZJU3	ASNS	Asparagine synthetase (glutamine-hydrolyzing)	1.700	0.053
P24797	ATP1A2	ATPase Na + /K + transporting subunit alpha 2	–1.420	0.053
Q90629	PRSS2	Serine protease 2	–2.500	0.053
O73885	HSPA8	Heat shock protein family A (Hsp70) member 8	–1.420	0.054
P62846	RPS15	Ribosomal protein S15	2.000	0.060
P26990	ARF6	ADP ribosylation factor 6	2.600	0.061
Q90593	HSPA5	Heat shock protein family A (Hsp70) member 5	–1.250	0.061
P30622	CLIP1	CAP-Gly domain containing linker protein 1	∞	0.062
P09540	MYL4	Myosin light chain 4	1.400	0.063
O75083	WDR1	WD repeat domain 1	3.000	0.063
P10184	SPINK5	Serine peptidase inhibitor, Kazal type 5	1.500	0.065
O59725	mic60	MICOS complex subunit Mic60	8.000	0.066
Q5ZL77	RIC8A	RIC8 guanine nucleotide exchange factor A	∞	0.066
P02675	FGB	Fibrinogen beta chain	1.800	0.068
Q90835	EEF1A1	Eukaryotic translation elongation factor 1 alpha 1	1.400	0.069
Q5ZLD7	VPS53	VPS53, GARP complex subunit	∞	0.071
P12276	FASN	Fatty acid synthase	1.600	0.072
E1C2P3	HSPA14	Heat shock protein family A (Hsp70) member 14	3.200	0.072
Q5ZJJ2	RPA1	Replication protein A1	–2.500	0.073
P02314	HMGN2	Non-histone chromosomal protein HMG-17	–2.500	0.075
Q5ZLN0	LRRC40	Leucine rich repeat containing 40	2.000	0.076
O93256	KRT19	keratin 19	9.700	0.077
Q9YHT2	SDHB	Succinate dehydrogenase complex iron sulfur subunit B	1.900	0.078
P51890	LUM	Lumican	–4.000	0.079
Q5ZLL5	COQ5	Coenzyme Q5, methyltransferase	1.800	0.084
P04354	CALB1	Calbindin 1	–1.600	0.086
Q5ZI72	HNRNPDL	Heterogeneous nuclear ribonucleoprotein D like	1.800	0.086
P02001	HBAD	Hemoglobin subunit alpha-D	1.800	0.088
P08251	ATP1B1	ATPase Na+/K+transporting subunit beta 1	–2.500	0.089
P09102	P4HB	Prolyl 4-hydroxylase subunit beta	–1.250	0.090
P12957	Cald1	Caldesmon 1	–1.250	0.094
P68139	ACTA1	Actin, alpha 1, skeletal muscle	3.200	0.096
P02612	MYL9	Myosin light chain 9	1.700	0.096
P26583	HMGB2	High mobility group box 2	–2.500	0.097
P08940	LECT2	Leukocyte cell derived chemotaxin 2	5.100	0.097
Q5ZKQ5	CASC4	Cancer susceptibility 4	–1.600	0.100
O13268	PSMA7	Proteasome subunit alpha 7	–1.420	0.100
Q5ZHX1	RAP1B	RAP1B, member of RAS oncogene family	1.400	0.100
P27003	S100A10	S100 calcium binding protein A10	∞	0.100

### Biological Functions

**FIGURE 3:**
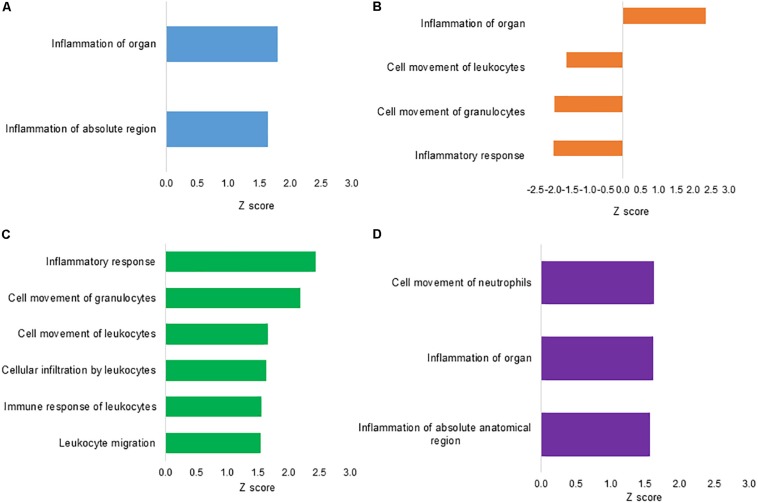
Downstream effects analysis based on differentially expressed proteins from ileal samples of broilers early exposed to lactic acid bacteria mixture (L), *Citrobacter freundii* (CF), or *Citrobacter* spp. (C2). Biological functions involved with inflammation and immune response processes of (**A**) L vs. S, following (**B**) CF vs. S, (**C**) L vs. CF, and (**D**) L vs. C2. The vertical axis shows the predicted biological function, while the horizontal axis shows the *Z* score calculated for the particular effect.

To assess the functional annotation associated with DEPs, the disease processes and cellular functions were predicted by the IPA approach. Figure 3 summarizes the major predicted effects on functional annotation coordinated to the inflammatory and immune responses. In L treatment, inflammation of organ (*Z* score = 1.80) and inflammation of absolute region (*Z* score = 1.65) were predicted to be activated in relation to S control group (Figure 3A). In CF, a predicted high activation *Z* score value was assigned only to inflammation of organ (*Z* score = 2.32). Biological functions associated with inflammatory response (*Z* score = −1.96), cell movement of granulocytes (*Z* score = −1.95) and leukocytes (*Z* score = −1.60) were predicted to be inhibited when compared to S control group (Figure 3B). There was no qualified prediction of downstream functional effects in C2.

To better understand the differential regulation of inflammatory and immune signaling response across treatments, the L protein expression metadata generated by IPA were further compared to CF and C2 profiles (IPA comparison analyses). As illustrated in Figure 3C, when L was compared to CF, the biological functional analysis predicted the activation of leukocyte migration (*Z* score = 1.54), immune response of leukocytes (*Z* score = 1.56), cellular infiltration by leukocytes (*Z* score = 1.64), cell movement of leukocytes (*Z* score = 1.67), cell movement of granulocytes (*Z* score = 2.19), and inflammatory response (*Z* score = 2.44). When examining L versus C2, there were predicted activation of inflammation of absolute anatomical region (*Z* score = 1.57), inflammation of organ (*Z* score = 1.62), and cell movement of neutrophils (*Z* score = 1.63; Figure 3D).

### Inflammatory-Related Proteins

**FIGURE 4:**
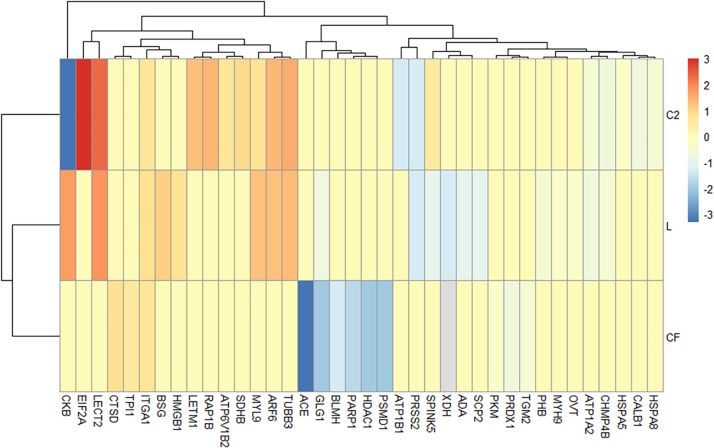
Differentially expressed proteins (DEPs) profiling identified by Ingenuity Pathway Analysis associated with the inflammatory annotation. **(A)** Heatmap plot represents the log2-fold changes of proteins expressed in ileal samples of broilers treated with lactic acid bacteria mixture (L), *Citrobacter freundii* (CF), or *Citrobacter* spp. (C2) in relation to control treatment. Orange shades represent underexpression, while blue shades indicate overexpression of the particular protein. Light gray cells indicate no expression. Hierarchical clustering in the rows is based on the protein composition similarity between treatments, while that in the columns is based on protein abundance.

Based on the IPA diseases and function annotation, the proteomic signatures associated with inflammatory downstream effects were identified (Figure 4 and Supplementary Table S5). Venn diagrams show the ileal DEPs related to inflammatory molecular functions (Supplementary Figure S1) in each *in ovo* treatment. The exposure to L *in ovo* downregulated the expression of eleven proteins (PRSS2, XDH, ADA, SCP2, SPINK5, ATP1A2, GLG1, PHB, MYH9, OVT, and ACE) and overexpressed eight (HMGB1, ITGA1, BSG, ARF6, MYL9, TUBB3, CKB, and LECT2). Similarly, there were nine downregulated (ACE, GLG1, HDAC1, PSMD1, PARP1, BLMH, PRDX1, TGM2, and PKM) and three upregulated DEPs (TPI1, ITGA1, and CTSD) found in CF versus S control. In the C2 treatment, the expression of eight proteins (CKB, ATP1B1, PRSS2, CALB1, CHMP4B, ATP1A2, HSPA8, and HSPA5) related to the inflammatory mechanism was downregulated. Likewise, seven DEPs (RAP1B, SPINK5, ATP6V1B2, SDHB, LECT2, TUBB3, MYL9, LETM1, ARF6, ITGA1, and EIF2A) were found to be upregulated in the ileum of C2-treated chicks. The Venn diagram showed that nine proteins (ATP1A2, TUBB3, LECT2, MYL9, PRSS2, CKB, ARF6, SPINK5, and CHMP4B) were present throughout L and C2 treatments, and only ITGA1 was shared across L, CF, and C2 (Supplementary Figure S1). It is also important to note that some of the above proteins were implicated in more than one process.

### Causal Network

Network analysis, derived from Ingenuity Knowledge Base, was drawn to determine the likely relevant causal relationships for changes in DEPs within the *in ovo* datasets. The top-enriched network in L treatment contained proteins associated with nucleic acid metabolism, skeletal and muscular development, and small molecule biochemistry (score, 44; 19 molecules, Supplementary Figure S2A). The DEPs identified in this network included ACP1, ACTA1, ACTR2, ADA, AGRN, ATP1A2, BSG, CKB, KIF2A, MX2, MYH9, MYL4, MYL9, P4HA1, PGAM1, RPS17, TARDBP, UBA5, and XDH.

**FIGURE 5:**
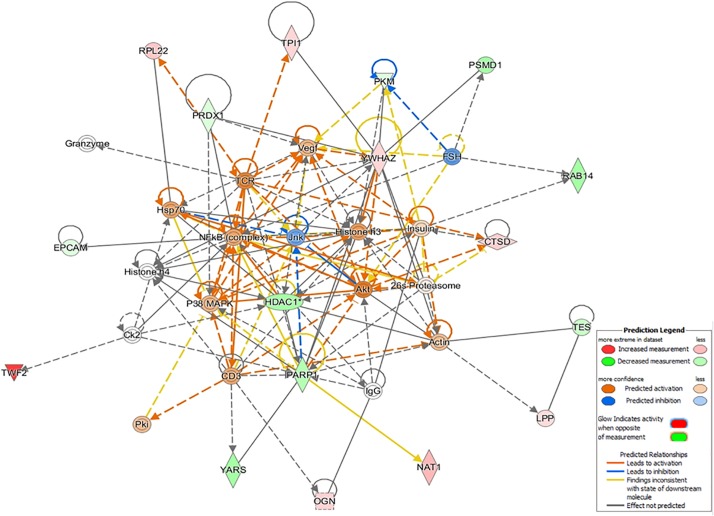
The top-enriched IPA causal network in *Citrobacter freundii* (CF) relates to cancer, organismal injury, abnormalities, and respiratory disease. The networks are built graphically as nodes (gene/gene products) and edges (the biological relationship between nodes). The red nodes represent the upregulation of proteins in the dataset, while green represents downregulation. The color intensity represents the relative magnitude of change in protein expression.

Interestingly, only in the CF dataset, the top-enriched network was composed of several proteins related to immunological and inflammatory functions, as well as the hub regulators Hsp70, nuclear factor-κB (complex), Vegf, and CD3. This network complex was characterized as cancer, organismal injury, abnormalities, and respiratory disease (42 score, 17 molecules, Figure 5), and the determined DEPs were CTSD, EPCAM, HDAC1, LPP, NAT1, OGN, PARP1, PKM, PRDX1, PSMD1, RAB14, RPL22, TES, TPI1, TWF2, YARS, and YWHAZ.

In C2, the most enriched network was linked to cellular function and maintenance, endocrine system disorders, and small molecule biochemistry (score 47, 20 molecules, Supplementary Figure S2B). This network consists of 20 proteins in our proteomic data set (ATP1A2, ATP1B1, ATP6V1B2, Cald1, CLIP1, HMGCS1, HSPA5, ITGA1, KRT19, LRRC40, LUM, MYL4, P4HB, PGAM1, PRSS2, RAP1B, RPS15, RPS4X, S100A10, and SDHB).

### Correlation Analysis

**FIGURE 6:**
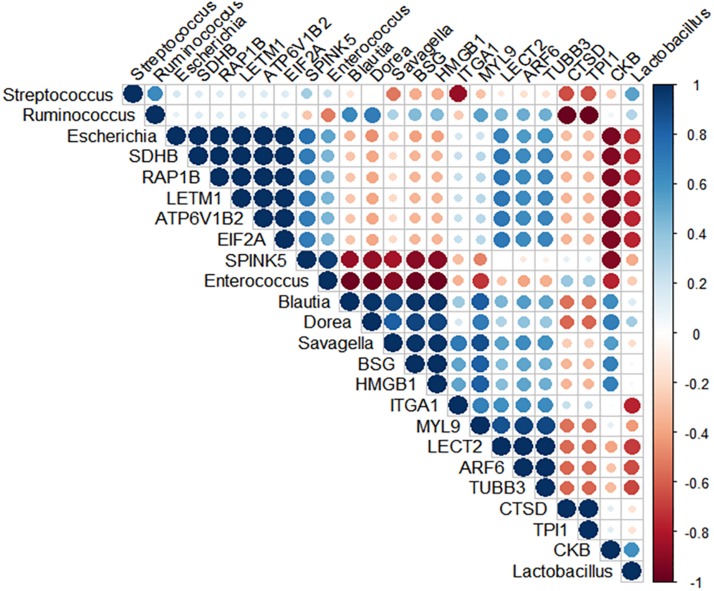
Spearman’s rank correlation matrix of the dominant microbial populations and inflammation-related proteins in the ileum of 10-day-old broilers. Strong correlations are indicated by large circles. The colors of the scale bar denote the nature of the correlation, indicating a perfect positive correlation (dark blue) and –1 indicating perfect negative correlation (dark red). *Candidatus Savagella* population was positively correlated with MYL9 (*R* = 0.95, *p* = 0.051) and ITGA1 (*R* = 0.95, *p* = 0.051), as well as *Enterococcus* abundance had a positive association with SPINK5 (*R* = 0.95, *p* = 0.051).

Spearman’s rank correlation was performed to search for potential positive relationships between the microbial composition and the intestinal inflammatory related-proteins (Figure 6 and Supplementary Table S6). Correlation analysis of the broilers’ intestinal proteome and bacterial abundance revealed a positive relationship between *Candidatus Savagella*, also known as segmented filamentous bacteria (SFB), and myosin light chain 9 (MYL9; *R* = 0.95, *p* = 0.051) and Integrin alpha-1 (ITGA1; *R* = 0.95, *p* = 0.051). In addition, *Enterococcus* was positively associated with serine peptidase inhibitor Kazal type 5 (SPINK5; *R* = 0.95, *p* = 0.051).

## Discussion

Given the magnitude of understanding the cross-talk between intestinal microbiota and host physiology, the impact of the early intestinal colonization on the microbiota in young broilers has previously been addressed by our lab (Rodrigues et al., 2019; Wilson et al., 2019). To comprehensively complement these previous studies, we used a proteomic approach to examine the ileal protein composition in response to the early exposure to L-based probiotics or *Enterobacteriaceae* strains. The findings presented here indicate that the intestinal pioneer colonization may modulate the immunological functions of young broilers.

Predicted function analyses showed enhanced annotation related to inflammation signaling in broilers *in ovo* treated with L (*Z* score = 1.80; Figure 3A) and CF (*Z* score = 2.32; Figure 3B). The prediction of inflammatory signaling found in L treatment was supported by the upregulation of a marker of inflammation, such as high mobility group box 1 (HMGB1; Yang and Tracey, 2010). Furthermore, treatment-specific biological functions found in L were associated with inflammatory response (Figure 3C). In general, the inflammatory response may be beneficial for the host as an acute and transient mechanism to mediate clearance of inciting agents in the GIT. Alternatively, the failure to control inflammation could inflict chronic and severe tissue damage (Xiao, 2017; Ptaschinski and Lukacs, 2018).

Currently, the predominant knowledge of intestinal inflammation in poultry is based on dysbiosis and mucosal barrier leakage studies (Kuttappan et al., 2015; Bielke et al., 2017; Ducatelle et al., 2018). These physiological conditions are primarily caused by the proliferation and colonization of pathogens in the intestine. However, the recent multiomics pipelines have identified commensal bacteria not only influencing metabolic and immune function but also triggering a state of tolerance with an inflammation-like response (Buffie and Pamer, 2013; Kogut et al., 2018). It has been proposed that SFB, a *Clostridiaceae* member, have a dominant effect on the mucosal immune system via stimulation of T cells, such as Th17, and increased proinflammatory cytokines and immunoglobulin A (IgA) production. Owing to the fact that colonization of SFB stimulates IgA release, it has been postulated as one of the mechanisms by which SFB might control pathogen overgrowth (Chen et al., 2018). On the other hand, the excessive immune reactions driven by SFB may also be accompanied by a physiological inflammation status (Ivanov et al., 2009; Ivanov and Littman, 2010; Chung et al., 2012; Buffie and Pamer, 2013). Our microbiome results previously published by Wilson et al. (2019) and Rodrigues et al. (2019) showed the succession of microbial communities colonization through the maturation of microbiota in chicks treated with L *in ovo*, with no evidence of potential pathogens overgrowth. Nonetheless, the previous findings showed a reduction in *Enterococcus* abundance and a particular prominent population of SFB in lower ileum of 10-day-old broilers in L treatment (Supplementary Table S1; Rodrigues et al., 2019). In this context, further action was taken to identify a potential microbial signature, which could be highly associated with the observed ileal inflammatory signaling. Spearman’s coefficient analyses revealed a strong positive correlation between SFB population and ITGA1 and MYL9. The protein MYL9 plays a crucial role in the complex system that regulates the contraction of smooth muscle. Recently, Hayashizaki et al. (2016) reported that MYL9 and MYL12 are functional ligands for CD69, suggesting that CD69-MYL9/12 interaction is also involved in recruiting activated cells to inflamed tissues. Indeed, high expression of MYL9/12 was related to sepsis-induced acute kidney injury, inflamed mouse airways, and patients with eosinophilic chronic rhinosinusitis (Wu et al., 2015; Hayashizaki et al., 2016). Similarly, Zhang et al. (2018) identified ITGA1 as an inflammatory-associated gene. Notably, we speculate that the high SFB population may be driving the predicted ileal mucosa proinflammatory status found in broilers early exposed to L. Nevertheless, the mechanism underlying SFB-mediated inflammation is yet unknown and must be further explored. Other groups have hypothesized that the interaction of commensal microbiota and young broilers during the development of the immune system could result in a transient inflammation without tissue damage. To the best of our knowledge, the link between SFB colonization and intestinal inflammation in broilers has not been proposed before. Future research is warranted to validate the SFB-associated proteomic signature generated from mass spectrometry. Such studies have the potential to develop markers of colonization and physiological effects mediated by SFB in human medicine and livestock.

Further support of early-life microbiota in programming immunological functions was shown by the biological annotation and causal network analyses. Treatment with L promoted a differential regulation of systemic immune processes in molecular profiles compared to CF and C2. The exposure of embryos to L enhanced the immune response function annotation associated with activation and trafficking of immune cells (Figures 3C,D), and the top-enriched network was related to skeletal growth (Supplementary Figure S2A). *Citrobacter* treatments, particularly CF, promoted inhibition of functions linked to immune cell migration and inflammatory response (Figures 3C,D, 5). Besides, based on the up- and downexpression pattern of proteins and the cause–effect relationships existing in Ingenuity Knowledge Base, a network related to injury and abnormalities in the organ was predicted in CF. This network is concerned primarily with the canonical nuclear factor-κB pathway, which is activated during the onset of inflammation (Lawrence, 2009). Our recent research has shown that the neonatal *Enterobacteriaceae* colonization mediated intestinal proteomic changes accompanied by inflammation in newly hatched chicks (Wilson et al., 2020). Chronic inflammation develops when the immune system is unable to clear a persisting insult, which generates a harmful environment and results in tissue impair (Meirow and Baniyash, 2017). Under all conditions, long-term inflammation can suppress immunity by decreasing immune cell numbers and function and/or increasing active immunosuppressive mechanisms (Dhabhar, 2009). Based on the cumulative findings, it is thought that the introduction of CF *in ovo* produced specific host–microbe interactions, which may have led to dysregulated immunity and immunosuppression by inducing long-term inflammation in 10-day-old broilers.

The maturation of the immune system starts in the first week of life in broilers (Crhanova et al., 2011), and although the immune maturation process can be driven by genetics and environmental conditions, the intestinal microbial composition has been identified to play a significant role in modulating immune responses (Crhanova et al., 2011; Chung et al., 2012; Arsenault et al., 2014; Gollwitzer and Marsland, 2015). Given these pieces of evidence, it is believed that manipulating the intestinal bacteria colonization with early-life exposure to L-based probiotics may modulate the development and maturation of immune functions of broilers. Madej and Bednarczyk (2016) and Pender et al. (2017) have reported that the modification of early-life intestinal microbiota through *Lactobacillus*-based probiotics *in ovo* stimulated an immunomodulatory effect in broiler chickens. In addition, the supplementation of host-tailored probiotics has shown to enhance colonization of ileal SFB, which may have contributed to confer immunostimulatory benefits for turkeys (Ward et al., 2019). Our analyses here show that the high colonization of SFB (Rodrigues et al., 2019) may be potentially associated with the enhancement of immune response in L-treated broilers at 10 days of age. These results increased the evidence that host-specific microbiota may drive the intestinal immune maturation (Ivanov et al., 2009; Crhanova et al., 2011; Chung et al., 2012; Buffie and Pamer, 2013; Hedblom et al., 2018).

In summary, the proteomics and bioinformatics analyses presented here suggest that despite shared predicted inflammation pathways by 10 days of age, triggers and inflammatory response were treatment specific in L and CF birds. Based on the microbiome profile previously published by Rodrigues et al. (2019), it is speculated that the high population of SFB in the ileum may be associated with the inflammation-like response found in L treatment, while the early intestinal colonization by *Enterobacteriaceae* may have caused a low-grade chronic inflammation in the intestine of CF birds. Supporting this, it was highlighted that proper immune function was dependent on specific intestinal microbiota. For instance, exposure of L-based probiotics may have shaped the development of immune functions, whereas the complexity of intestinal microbiota caused by early colonization of *Enterobacteriaceae* strains may have dysregulated the immunological response in 10-day-old broilers.

## Data Availability Statement

The mass spectrometry proteomics data have been deposited to the ProteomeXchange Consortium via the PRIDE partner repository with the dataset identifier PXD015504.

## Ethics Statement

The animal study was reviewed and approved by the Institutional Animal Care and Use Committee (IACUC).

## Author Contributions

DR, MT, KW, AD, KC, and WB carried out the project. DR performed analyses, interpreted the results, and wrote the manuscript in consultation with LB and WB. All authors contributed to experimental design, discussed the results, and commented on the manuscript.

## Conflict of Interest

The authors declare that the research was conducted in the absence of any commercial or financial relationships that could be construed as a potential conflict of interest.

## Footnotes

1 http://www.ingenuity.com
