# Supplementary material for: A Proteomic View of the Cross-Talk Between Early Intestinal Microbiota and Poultry Immune System
Source: Front Physiol. 2020 Feb 13;11:20. doi: 10.3389/fphys.2020.00020 (PMC7031415; doi:10.3389/fphys.2020.00020)
Supplement: Supplementary file 1 [file Data_Sheet_1.pdf]

# *Supplementary Material*

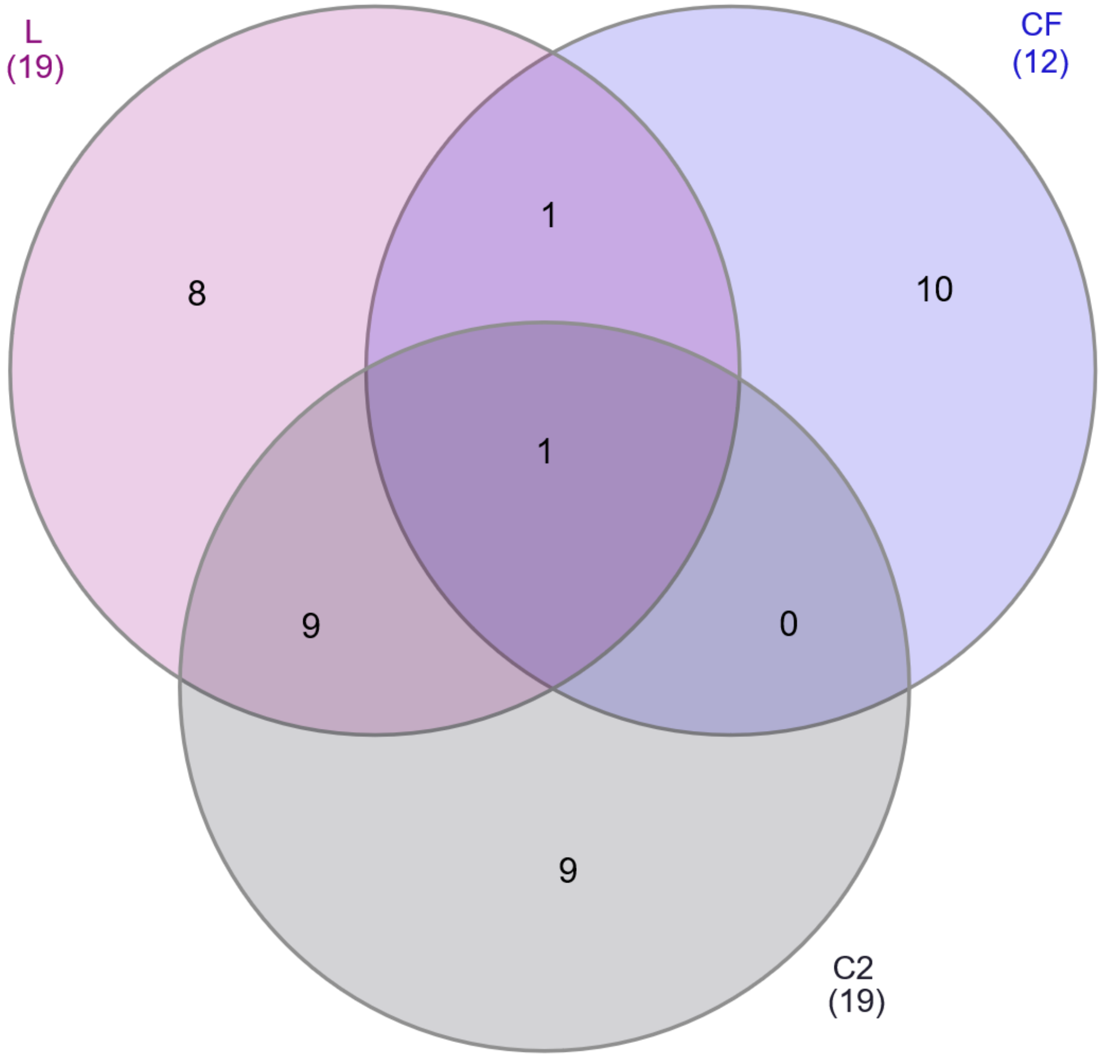


**Supplementary Figure 1.** Venn diagram showing differentially expressed proteins (DEPs) related to inflammation signaling among treatments.

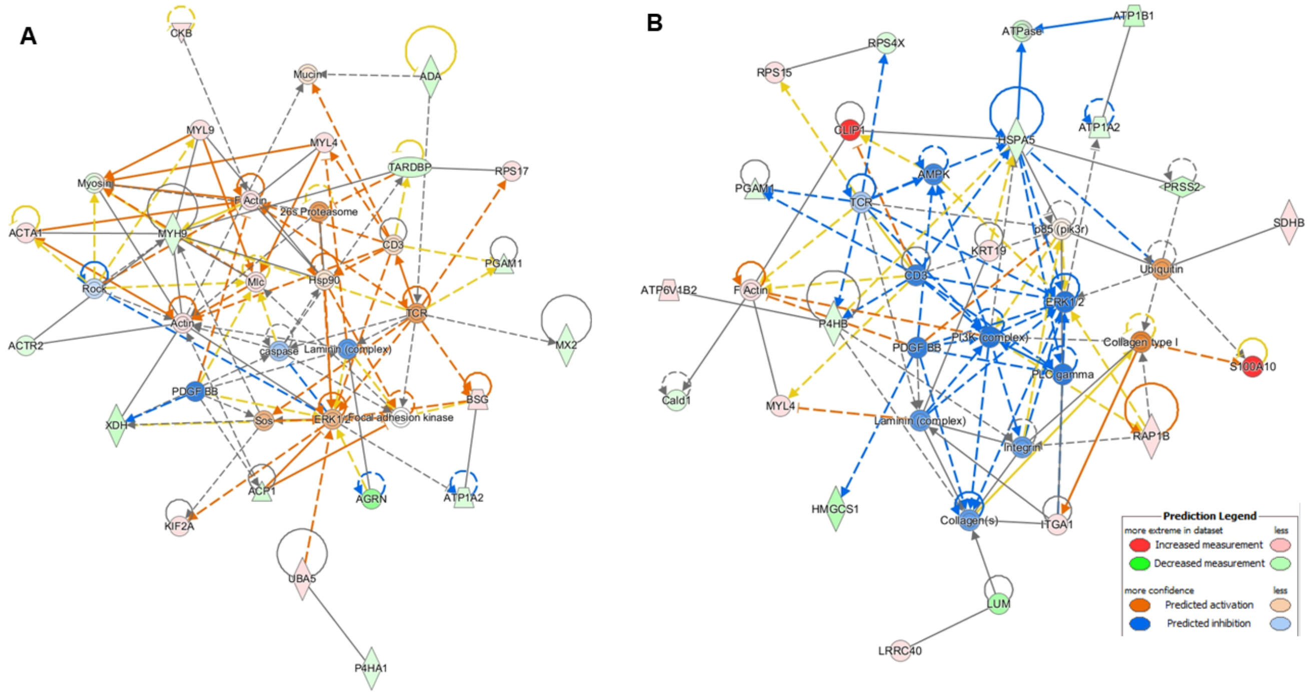


**Supplementary Figure 2**. The top-enriched causal networks. (**A**) Network generated in lactic acid bacteria mixture (L) dataset reveals biological functions associated with nucleic acid metabolism, skeletal and muscular development, and small molecule biochemistry. (**B**) Differentially expressed proteins in the *Citrobacter* spp. (C2) dataset discloses a network directly related to cellular function and maintenance, endocrine system disorders and small molecule biochemistry. The networks are built graphically as nodes (gene/gene products) and edges (the biological relationship between nodes). The red nodes represent upregulation of proteins in the dataset, while green, downregulation. The color intensity represents the relative magnitude of change in protein expression.
